# Update in the methodology of the chronic stress paradigm: internal control matters

**DOI:** 10.1186/1744-9081-7-9

**Published:** 2011-04-27

**Authors:** Tatyana Strekalova, Yvonne Couch, Natalia Kholod, Marco Boyks, Dmitry Malin, Pierre Leprince, Harry MW Steinbusch

**Affiliations:** 1 Department of Neuroscience, Faculty of Health, Medicine and Life Sciences, Maastricht University, Maastricht, Netherlands; 2 Center of Environmental Biology, Faculty of Sciences, Lisbon University, Lisbon, Portugal; 3 Department of Pharmacology, Oxford University, Oxford, UK; 4 Institute of General Pathology and Pathophysiology, Russian Academy of Medical Sciences, Moscow, Russia; 5 Northwestern University, Feinberg School of Medicine, Lurie Cancer center, Chicago, IL, USA; 6 GIGA-Neuroscience, University of Liege, Liege, Belgium

**Keywords:** animal model of depression, chronic stress, sucrose test, anhedonia, antidepressant treatment, gene expression profiling, neuroinflammation, mouse

## Abstract

To date, the reliability of induction of a depressive-like state using chronic stress models is confronted by many methodological limitations. We believe that the modifications to the stress paradigm in mice proposed herein allow some of these limitations to be overcome. Here, we discuss a variant of the standard stress paradigm, which results in anhedonia. This anhedonic state was defined by a decrease in sucrose preference that was not exhibited by all animals. As such, we propose the use of non-anhedonic, stressed mice as an internal control in experimental mouse models of depression. The application of an internal control for the effects of stress, along with optimized behavioural testing, can enable the analysis of biological correlates of stress-induced anhedonia versus the consequences of stress alone in a chronic-stress depression model. This is illustrated, for instance, by distinct physiological and molecular profiles in anhedonic and non-anhedonic groups subjected to stress. These results argue for the use of a subgroup of individuals who are negative for the induction of a depressive phenotype during experimental paradigms of depression as an internal control, for more refined modeling of this disorder in animals.

## Introduction

Depression is projected to become the second most common cause of disability worldwide by 2015. Depression as a major health issue is illustrated by its death-toll, which currently claims more lives per year than road-traffic accidents [1-4]. At the same time, there is an obvious need for an improvement in the treatment of depression, as up to 45% of depressed patients do not show improved mood after advanced therapy and 15% of patients do not respond to any antidepressant therapies [5]. The Diagnostic and Statistical Manual, Fourth Edition (DSM-IV) defines depression by the presence of at least one of two core symptoms: *anhedonia*; a decreased ability to experience pleasures, and *depressive mood*; lasting minimally 2 weeks [6,7]. Since anhedonia, on the one hand, is a cardinal phenomenon of depressive disorders, and on the other, can be evoked in rodents, the hedonic deficit might be considered as a primary feature to be addressed in pre-clinical models of depression. Coping and cognitive deficits, low exploratory motivation, circadian and sleep disturbances, aggressive and anxiety traits, decreased sexual and increased submissive behaviour, social avoidance, deterioration of the coat state and other changes, which can be evoked in animals, with some considerations [8] are regarded as parallels of subsidiary depressive symptoms [9,10].

The aim of this review is to analyze the major methodological drawbacks in mouse models of depression with a focus on its principal feature, anhedonia, in a chronic stress paradigm, and to share with the reader several procedural modifications resulting from our own experiences with a chronic stress model in C57BL/6N mice. We believe that the changes to methodology proposed here provide important advances in modelling the neurobiological basis of depression in rodents and that their implication can help develop more effective therapeutic strategies.

### Challenges in modelling depression and anhedonia using chronic stress paradigms

The chronic stress paradigm is considered to have a greater aetiological relevance and face validity in mimicking depression than other animal models, and therefore has become one of the most broadly used pre-clinical paradigms of this disorder [11-14]. The first experiments using this model were undertaken by Katz and colleagues in rats, and involved rotating stressors applied over 21-days [15]. These procedures were later modified by Willner resulting in higher length of experiment and lower severity of stressor [16]. These experiments resulted in a decreased preference for, and intake of palatable solutions such as sucrose or saccharine. This was defined as stress-induced anhedonia. This anhedonic state was accompanied by an increase in the thresholds required for intracranial self-stimulation and was reversed by anti-depressants, but not by neuroleptics or anxiolytics [13,17]. By now, a number of variants of chronic stress procedures had been proposed in both rats and mice and had been shown to evoke, in addition to anhedonia, the subsidiary depressive-like features mentioned above [18-31].

Regrettably, a number of studies revealed inconsistencies in the induction of hedonic deficit in chronic stress models in both rats and mice [32-35]. For example, in one study, Wistar and PVG hooded rats were subjected to chronic mild stress; stressed animals of both lines showed "unreliable" decreases in sucrose intake, which were "inconsistent" over time. None of stressed animals showed a decrease in the intracranial self-stimulation, evaluated by 50% of maximal response rate in the rate/frequency function [36]. Problems with reproducibility could also be due partly to the limited accuracy of the sucrose test, which, in its current state, does not have sufficient resolution to discriminate between anhedonic and non-anhedonic individuals within a stressed population [37-39]. In addition, some of multi-disciplinary studies, using anhedonic chronic stress models of depression, have resulted in abstruse and contradictory outcomes, and failed to define a consistent molecular, neuroanatomical and physiological phenotype in either rats or mice. Data on their locomotion, anxiety, exploration, and other behaviours often demonstrated paradoxical and conflicting behavioural changes; many of them showed discrepancies between the behavioural phenotype of chronically stressed animals and human symptoms of depression. Together, controversies with reproducibility of stress-induced anhedonia, defined by sucrose preference data and identification of biological correlates of depression greatly limit the value of this method to model pre-clinical depression [40].

Apart from methodological problems, application of the chronic stress approach has encountered some conceptual drawbacks. The most obvious is that in previously proposed models, all effects observed in groups of chronically stressed animals with signs of a decreased sensitivity to reward, are attributed to an hedonic deficit. It is important to note that stress alone can evoke a number of physiological alterations, which are not associated with a depressive-like behavior and anhedonia. With the originally proposed models and their analogues it was not possible to correlate findings obtained in chronically stressed animals with anhedonia, thus, specific biological correlates of hedonic deficit could not be addressed.

Studies with our new model of stress-induced anhedonia suggest that unresolved methodological difficulties in measuring behaviour in chronically stressed animals may be the origin of the above problems. Here, we present data obtained across several experiments, which reveal the major sources of behavioural artifacts in chronic stress mouse models of depression. These data enable us to propose several changes to the accepted methodology which are validated by both behavioural and molecular correlates of anhedonia.

### Anhedonia is exhibited by a subgroup of animals in chronic stress paradigms

**Figure 1:**
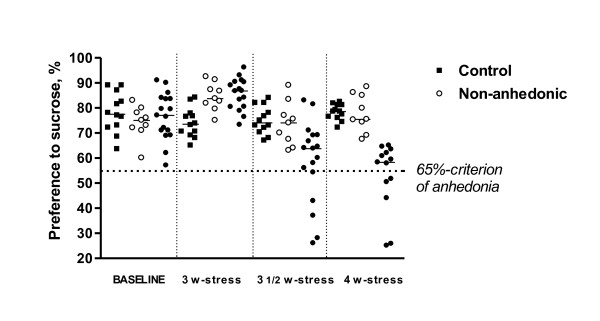
**Chronic stress results in decreased sucrose preference in a subgroup of mice**. After initial increase in sucrose preference on the 3rd week of stress, by the termination of a 4-week stress, a cohort of mice display a prominent decrease in sucrose preference. Mice subjected to chronic stress were split into anhedonic and non-anhedonic subgroups according to the criterion of 65% preference for sucrose solution (see the text), Reproduction of this material is permitted by Macmillan Publishers Ltd.

Numerous findings show a remarkable inter-individual variability in animals' responses to stress [9,13,41-43]. Using a principle of the isolation of responders and non-responders, we have established a mouse model of stress-induced anhedonia with an internal control for the effects of stress alone [44]. A four week chronic stress paradigm, comprised of exposing male 3-months-old C57/BL6N mice to tail suspension and restraint stress in different procedural variants resulted in a decrease of preference to 1% sucrose solution by ≤ 65% in 50-70% of animals [[44-49]; Figure 1]. It was shown that sucrose preference is similar in control, non-anhedonic and anhedonic mice before the onset of stress. At the end of the stress induction period sucrose preference and intake markedly decrease and are replaced by an increase in water consumption.

Recent studies in chronically stressed mice and rats have shown similar partial depressive-like outcomes ([21-25]; see Table 1). Resilience or susceptibility to stress-induced anhedonia and/or other physiological parallels of depressive-like state in newly developed models was found to correlate with distinct regulation of mesolimbic dopamine circuits, excitability of dopamine neurons of ventral tegmental area [21,50], activity of the medial prefrontal cortex, nucleus accumbens and amygdala [21,51], differential expression of neuropeptide Y, transcription and factors markers, immediate early genes, a dysfunction in the GABAergic system and AMPA-mediated transmission [21,23,51-57], as well as changes in learning capacities including working memory [56,57] and hippocampal neurogenesis [58].

**Table 1:** Partial depressive-like outcomes in chronic stress models of depression

Stress	Laboratory strain	Measure of a depressive-like state	Susceptibility rate	Reference
6-week CMS	Wistar rats	Low sucrose preference	51.5%	[25]

5-week social stress	CD1 mice	Increase of CORT, Low sucrose preference	20%	[24,57]

7-week CMS	Wistar rats	Low sucrose intake	50-70%	[23,54-56]

10-day social defeat	C57BL/6J	Social avoidance, Low sucrose preference	50-70%	[21,50-53,58]

7-week unpredictable CMS	Eleven common mouse strains	Coat deterioration, Low sucrose preference	Vary in different strains	[22,41,60]

Recent studies identified susceptible and resilient subpopulations of chronically stressed mice defined by anhedonia occurrence in a sucrose test, deterioration of a coat state, or an increase in basal CORT levels.

The inter-individual variability in susceptibility or resilience to stress-induced decreases in sucrose preference observed in these, and our, paradigms suggest that this phenomenon is typical, even for inbred lines of animals [21,56,59-63]. A variety of potential mechanisms may underlie this phenomenon: 1) pre-natal and early environmental factors [64] epigenetic mechanisms consisting in DNA and chromatin modifications, histone acetylation and methylation [65,66] and switches and error-prone DNA replicates [67]; 3) large-scale organization of gene expression levels and multigenic trait mechanisms [68,69], 7) posttranslational regulation of proteins [70]. Generally, preservation, even in genetically homogenious lines of animals, of inter-individual variability of physiological parameters allows one to develop hypotheses regarding its importance as a biological factor of adaptation and survival, especially relevant in a stressful environment, which may be mediated by specific biological mechanisms.

### Physiological features of stress and stress-induced anhedonia in mice

Original protocols using C57BL/6N mice for studying emotionality, locomotion, exploration, cognition and other functions [38,71,72] were modified in order to preclude behavioural artifacts in testing chronically stressed mice (see below). So-called "mild" testing conditions applied with these modifications, were aimed at the reduction of stress in animals during their testing and therefore preventing the occurrence of stress-induced hyperlocomotion. Behavioural features of anhedonic and non-anhedonic mice are summarized in the Table 2. In contrast to non-anhedonic animals, anhedonic mice revealed increased floating during the forced-swim test and immobilization behaviour in the tail suspension test, reduced exploration of a novel environment and object, impaired hippocampal-dependent performance in the step-down passive avoidance, Y-maze and contextual fear conditioning tasks, and disrupted burrowing behaviour in the food displacement test. A development of the latter deficit, as well as of behavioural despair and disruption of contextual learning were precluded by chronic antidepressant treatment in our model [[46], Tokarski et al.: Impaired hippocampal plasticity in mice with hedonic deficit, induced by chronic stress, *unpublished*)]. The above listed changes in behaviour are considered as analogues of those seen in patients, diminished interest in novelty, cognitive decline and impairment of declarative memory [6,7]. These symptoms were mimicked in pre-clinical studies, many of which revealed the curative effects of antidepressants[9,10,13].

**Table 2:** Physiological correlates of stress and stress-induced anhedonia in our model

Physiological variable	Anhedonic Changes vs. control	Non-anhedonic Changes vs. control	Reference
**1. Floating in forced swim test**	Increased	Not changed	[38,44,45]

**2. Immobilization in tailsuspension test**	Increased	Not changed	[39,47]

**3. Novelty exploration**	Decreased	Not changed	[44]

**4. Burrowing behaviour**	Decreased	Increased	[38,39]

**5. Contextual memory in passive avoidance**	Decreased	Not changed	[38]

**6. Contextual fear conditioning**	Decreased	Not changed	[Tokarski et al. Impaired hippocampal plasticity in mice with hedonic deficit, induced by chronic stress (unpublished)]

**7. LTP in the CA1 area of the hippocampus**	Disrupted	Not changed	[Tokarski et al. Impaired hippocampal plasticity in mice with hedonic deficit, induced by chronic stress (unpublished)]

**8. REM sleep**	Increased	Not changed	[46,48]

**9. Home cage activity during dark phase**	Increased	Not changed	[47]

**10. Anxiety-like behavior in O-maze and dark-light box**	Increased	Increased	[38,44,108]

**11. Open field locomotion under modest lighting**	Increased	Increased	[38,44,108]

**12. Aggressive behavior**	Increased	Increased	[[95], unpublished data]

**13. Auditory fear conditioning**	Not changed	Not changed	[Tokarski et al. Impaired hippocampal plasticity in mice with hedonic deficit, induced by chronic stress (unpublished]

**14. Body weight**	Decreased	Decreased	[38,39,44-49]

Eight out of fourteen evaluated physiological variables had differential changes in anhedonic and non-anhedonic animals, as compared to control. Remaining parameters were either not altered in these groups, or were changed in the same direction, suggesting that not all physiological consequences of stress can be attributed to anhedonia in the employed model.

As well as behavioural changes, we found specific physiological abnormalities in anhedonic animals; a lack of ex-vivo LTP induction capacity in the CA1 area of the hippocampus during high frequency stimulation protocols, elevated duration of REM sleep and home cage hyperlocomotion (Table 2). Chronic treatment with an SSRI precluded stress-induced disruption of LTP induction in hippocampal slices. This data correlates with human studies showing that impaired hippocampal function and synaptic plasticity are sensitive to antidepressant treatment [73,74]. The augmentation of REM sleep and disturbances in diurnal rhythms observed in clinical studies and animal models of depression are thought to be characteristic of clinical depression [3-7,18,20]. In particular, depressed patients exhibit a high percentage of REM sleep when compared to other neuropsychiatric disorders with shared pathophysiology.

At the same time, many changes in animals were found to occur in stressed animals irrespectively of the presence of hedonic deficit: hyperlocomotion observed in brightly illuminated open field, hypoactivity in the dark open field, increased scores of anxiety-like behaviour in the dark/light and elevated O-maze tests, high scores of aggressive behaviour and loss of body weight (Table 2). Increased scores of anxiety, aggressive behaviour, locomotor inhibition and behavioural invigoration are also well documented in humans and are thought to be the result of stressful experiences [3-7,28,31,75].

In the present model, stress-induced loss of body weight, high scores of anxiety and locomotor diturbances are comparable in anhedonic and non-anhedonic mice that might be regarded as an indication of similar impact of the stress in these animals. Data concerning anxiogenic-like changes in the O-maze in C57BL/6J mice with and without social avoidance and anhedonia evoked by social defeat stress [21] are in line with our findings. In this study, no difference in body weight between two groups was observed at the time point of experiment, when signs of elevated depressive- and anxiety-like behaviour were revealed in stressed animals. The CD1 mice, either resilient or susceptible to chronic social stress, as measured by changes of basal CORT levels and sucrose preference, showed no alterations in body weight [57]. In studies on other mouse lines, a manifestation of stress-induced depressive-like features correlated with a loss of body mass [13,23,41,60]. This suggests that species-specific differences across different strains of laboratory mice may underlie a distinct relationship between anhedonia and body weight. In line with our results, both anhedonic and non-anhdonic Wistar rats in the chronic stress model were found to have similarly decreased body weight [25]. We believe that the absence of differences in body mass in our model between anhedonic and non-anhedonic mice may be caused by the 'ceiling effect' of stress on physical parameters in a specific strain. Similar values of body weight in subgroups of stressed mice can be an important factor, which prevents significant confounds in the behavioural comparison of anhedonic and non-anhedonic mice. Should body weight vary between these groups, their comparison in behavioural tests based, for example, on the measurement of liquid intake and foot shock application is likely to be compromised by distinct metabolic features and a response to an electrical stimulation.

The latest literature suggests a link between common inflammatory factors, loss of body weight and sickness behaviour - a state which is related to anhedonia. In the light of recent identification of inflammatory factors, which underlie both sickness behaviour and depression [76], it can be speculated that stress-induced decreases in body weight, in addition to classical mechanisms of hormonal secretion and sympathetic activation, might be mediated by activated inflammatory pathways involved in the pathogenesis of anhedonia. Of note, in our model stressed, anhedonic mice showed disrupted burrowing behaviour that we believe to be related to both lesions of the dorsal hippocampus and sickness behaviour [38,39,77]. While, in our studies, both susceptible and resilient animals showed a robust decrease in body weight, a potential contribution of inflammatory factors to this consequence of stress could be an interesting question to be addressed experimentally.

Interestingly, elevated anxiety was found in stressed animals irrespective of the occurrence of hedonic deficit, supporting the clinical view of the comorbidity of anxiety and depression, where core pathogenetic mechanisms are thought to be distinct [78,79]. These distinct mechanisms are used to separate anxiety from anhedonia in animal models of depression. Of importance, a stress-induced anhedonic state was found to be selectively associated with depressive-like features, which were not observed in stressed non-anhedonic animals. In contrast, pathological changes that are common for a broad spectrum of stress-related disorders besides depression, e.g., behavioural invigoration, and high anxiety are shared between stressed mice with and without depressive-like features. The fact that anhedonic mice display depressive-like traits which are not detected in the non-anhedonic group speak in favour of the face validity of the employed model as a model of depression at the first instance, and argue for the adequacy of the latter group as a proper control for the effects of stress that are not associated with a depressive-like state. Several groups who have employed a similar approach have obtained comparable outcomes that further support our original findings concerning differential physiological profiles of animals resilient and susceptible to stress-induced depression (Table 3).

**Table 3:** Features of resilience and susceptibility to stress-induced depressive state in animal models

Physiological variable	Susceptible	Resilient
**Sucrose intake/preference**	Decreased [21-25]	Not changed [21-25]

**Behavioural despair**	Increased [23,25] Not changed [21]	Not changed [21,23,25]

**Social avoidance**	Increased [21,22]	Not changed [21,22]

**Novelty exploration**	Decreased [21,22] Increased [25]	Not changed [21,22] Increased [25]

**Contextual memory**	Decreased [24] Not changed [23]	Not changed [23,24]

**Working memory**	Decreased [24]	Not changed [24]

**Coat state and self-grooming**	Impaired [22,24]	Not changed [22,24]

**Anxiety-like behavior**	Increased [21,22,24] Not changed [25]	Increased [21,22,25] Not changed [24]

**Body weight**	Decreased [21,22,25] Not changed [21-24]	Decreased [21,22,25] Not changed [21-24]

**Social hyperthermia**	Increased [21]	Not changed [21]

**Stress-induced polydipsia**	Increased [21]	Not changed [21]

**Circadian amplitude**	Dereased [21]	Not changed [21]

**Conditioned place preference**	Increased [21]	Not changed [21]

Newly available models of depression which differentiate resilient and susceptible cohorts of animals enable a distinction of their physiological profiles. Original reports describing animal paradigms are quoted; in some cases dependently on the measure taken and the strain used the outcome varied within the same paradigm.

### Identification of molecular correlates of stress and stress-induced anhedonia

Altered functional activity within the hippocampal formation is thought to be important hallmark of stress and a depressive-like state [80,81]. Utilizing the hippocampi of control versus stressed anhedonic and non-anhedonic mice, gene expression profiling has been performed using Illumina technology (IntegraGen, Evry, France) and the Ingenuity Pathway Analysis program (Ingenuity Systems, Redwood city, CA, USA). Anhedonic and non-anhedonic mice displayed substantial changes in clusters of genes involved in specific biological functions important to sustain various CNS processes (Table 4). While a detailed analysis of the likely outcomes of the affected functions on hippocampal physiology has not been performed, it is interesting to note that some of the observed changes in anhedonic mice are part of categories consisting of negative or inhibitory processes. These include such clusters of functions classified with the Ingenuity Pathway Analysis program, as impaired psychological processes, coordination and memory, pre-pulse inhibition and long term and synaptic depression. Genes related to neurological disease and disorders, and to neurodegeneration are also affected. For the non-anhedonic mice, the functions with higher gene expression are important in such processes as biogenesis, formation, growth and development of cells, neurites and vesicles. Positive behaviour and psychological processes, such as cognition, learning and LTP are also affected.

**Table 4:** Comparison of gene expression in anhedonic and resilient stressed mice versus non-stressed controls

Category	Function	Anhedonic			Resilient		
		
		# Genes Up	# Genes Down	p-value	# Genes Up	# Genes Down	p-value
**Cellular Assembly and Organization**	transport of vesicles			N.S.	41		4.22E-07
	biogenesis of cytoskeleton			N.S.	35	3	5.41E-06
	morphogenesis of neurites			N.S.	33		7.25E-06
	formation of vesicles			N.S.	25		2.04E-05
	formation of filaments	11	13	4.47E-05			N.S.
	formation of neurites	4	8	8.47E-05			N.S.

**Behavior**	cognition	9	6	2.62E-03	57	1	2.97E-06
	behavior	30	16	1.29E-04			N.S.
	learning			N.S.	48	1	2.13E-05
	psychological process of mice	23	10	2.49E-04			N.S.
	memory	10	5	3.01E-04			N.S.
	exploratory behavior of mice	6	1	1.80E-04	14		5.37E-04

**Cellular Movement**	movement of brain cells			N.S.	30	1	3.40E-06
	migration of brain cells			N.S.	29	1	4.59E-06

**Nervous System Development and Function**	development of neurites	18	4	1.67E-05	63		6.50E-06
	development of axons	11	4	3.10E-05	34		1.35E-03
	long-term potentiation	10	5	2.54E-03	50	3	1.60E-05
	long term depression	6	3	5.95E-04			N.S.
	synaptic transmission	13	6	2.77E-05	50		1.29E-04
	prepulse inhibition of mice	8	2	2.66E-06			N.S.
	coordination of mice	5	2	9.73E-05			N.S.
	memory	10	5	3.01E-04			N.S.
	spatial memory of mice	5	3	3.96E-04			N.S.
	neurological process	25	19	4.65E-08	107	4	1.30E-03
	neurotransmission	17	7	6.95E-07	57		1.30E-04

**Neurological Disease**	seizures			N.S.	35	1	1.11E-04
	dyskinesia			N.S.	76	2	1.86E-04
	Huntington's disease	18	11	2.11E-06	75	1	2.06E-04
	neurological disorder	42	41	2.36E-06	258	11	5.50E-03
	neurodegeneration of neurons	8	3	2.75E-04	27		6.10E-04

Gene expression data sets containing gene identity and corresponding expression values from an Illumina Technology analysis have been analyzed using the Ingenuity Pathway Analysis program. Out of 4795 eligible genes, the analysis generated lists of the most significant biological functions that are likely to be affected in anhedonic stressed or resilient stressed mice hippocampi, respectively. For selected detailed functions, the numbers of up-regulated and down-regulated genes are shown, as well as the significance score of the change in expression calculated as p-value of a right-tailed Fisher exact test. N.S. indicates a non-significant test (p-value > 1E-02)

Another study with our model revealed an overproduction of the enzyme COX-2 in the hippocampus of anhedonic that was not observed in the non-anhedonic group (Strekalova and Steinbusch, *in preparation*). Pre-treatment with a pharmacological blocker of this enzyme with food carried out through the entire period of chronic stress elicited similar antidepressant-like effects to those, which were evoked by citalopram in this study (Table 5). Both drugs prevented a stress-induced decrease in sucrose intake and preference, behavioural despair and impairment of cognitive deficit in the fear conditioning paradigm. These data are in line with growing evidence that along with monoamines, factors of inflammation play a crucial role in the mechanisms underlying anhedonia and depression [76,82-84]. Recently, it has been established that pro-inflammatory factors induce not only symptoms of behavioural sickness which resemble an anhedonic state, but are also implicated in the pathogenesis of major depression. We believe that altered production of COX-2 specifically, and the pharmacological sensitivity of the main read-outs in our model, can be considered as an evidence for such a link.

**Table 5:** Effects of COX-2 inhibitor in mice subjected to chronic stress

Changes versus non-stressed control	Stressed drug naive	Stressed Citalopram-treated	Stressed treated with COX-2 blocker
Sucrose preference	↓	**↔**	**↔**

Sucrose intake	↓	**↔**	**↔**

Floating behavior	**↑**	**↔**	**↔**

Contextual fear conditioning	↓	**↔**	**↔**

Parameters of anxiety in elevated O-maze	**↑**	**↔**	**↑**

Percentage of anhedonic mice	**above 60%**	**below 25%**	**below 25%**

Chronic treatment with a COX-2 inhibitor during the course of chronic stress counteracted the development of hedonic deficit, behavioural despair, increased anxiety and cognitive impairment in a similar manner to citalopram. Arrows indicate an increase in the parameter (↑), a decrease (↓), or lack of significant changes in comparison to non-stressed control mice (↔).

Together, our data suggest distinct molecular correlates of states of stress and stress-induced anhedonia in a proposed model. Similarly, studies in a social defeat paradigm in C57BL/6J mice demonstrated that susceptible and resilient individuals, which are distinct in scores of social avoidance and sucrose intake and preference, have differential levels of immediate early genes Arc and Zif268 in the frontal cortex, BDNF in the hippocampus and the ventral tegmental area and DeltaFosB in the nucleus accumbens [21,50-53]. Studies in Wistar rats showed distinct expression patterns of BDNF and vascular endothelial factor in the hippocampus [23]. Differential expression of AMPA receptors in the dorsal hippocampus was revealed between resilient and susceptible individuals in a social stress model in outbred mice [57]. Further studies are required to address key pathogenetic gene expression factors of resilience and susceptibility to a depressive syndrome precipitated by stress; such studies are under way.

### Limitations of the sucrose preference test in assessing anhedonia in chronic stress paradigms

Several behavioural paradigms are currently used to measure sensitivity to reward in rodent chronic stress models. These include consumption of palatable solutions, progressive ratio responding, intracranial self-stimulation, novel-object place conditioning and conditioned place preference [36,61,85-87]. The sucrose/saccharine consumption free-access paradigm is probably the most extensively used method, as it is not too labour- or time-intensive, has high throughput and aritifacts related to learning, anxiety and locomotion are minimal with this model. Decreased intake and/or preference for palatable solutions is an overall validated behavioural measure of hedonic deficit [9-13,87]. Insufficient accuracy of the sucrose test in mice is, however, one of the key difficulties in measuring behaviour in chronic stress models of depression [14,32,38,39]. In mice, the sucrose test can typically reveal the differences between groups, but not between individual animals, and is generally considered to result in more variable outcome than in rats. A variety of sucrose test protocols have been proposed to overcome these inconveniences [88-93].

The species-specific physiological features of drinking behaviour and physical artifacts in its evaluation are believed to underlie the insufficient resolution of the sucrose test in mice [14,32,35,39]. In a series of experiments, we have identified factors of drinking behaviour in C57BL/6N mice, which may essentially interfere with the outcome of the standard free-choice, two-bottle sucrose test paradigm (Table 6). First, we found that individual mice of the tested strain showed a preference for drinking at one or the other corner of the cage. This preference was not related to any obvious external factors, e.g., source of noise or lighting in the room, as consumption of highly concentrated sucrose solution depended on its localization either in the preferred or non-preferred bottle position. Housing the mice with water on both sides of the cage abolished the side preference in drinking behaviour in some but not all animals. Switching the position of the bottles containing water or sucrose solution halfway through the test minimalised this confounding factor. Second, in C57BL/6N mice, one-bottle water tests revealed individual daily drinking patterns, specifically mice showed peaks in drinking behaviour during the 10-hour test period. Interestingly, temporal patterns in water intake correlated with mean volume of consumption. For example, animals with a peak in water intake 7.5 h after the beginning of the active phase of the animals' cycle had significantly higher values of total water intake than any other group. These data suggest that when analyzing drinking behaviour and sucrose preference, experiments should cover the periods of maximal liquid intake in all animals, as well as taking into account the relative parameters of preference in choice paradigms, rather than of absolute intake values. In addition, absolute consumption of palatable liquids can often be confounded by changes in the consumatory behaviour induced by deprivation of food and water, acute effects of stressors, altered metabolic needs in calories and water [32,34,35,37]. The greater variation in values of absolute intake of sucrose solution in comparison with those of sucrose preference, as shown by significant differences in variation [38,85,87], additionally define sucrose preference as a parameter, which is least influenced by animals' individual drinking patterns.

**Table 6:** Factors of potential confounds in a free drinking two-bottle sucrose test

Own behavioral data	Source of confounds	Preventing of confounds in testing	References
Sucrose solution intake is affected by a position of the bottle on preferable or non-preferable side. Weeks of housing with two bottles does not abolish side preference in drinking behaviour	Side preference in drinking	Switching of the bottles in a middle of the test	[21,22,24,38,47,92]

Individual patterns of absolute water intake in a 10-h test	Large individual variability in daily drinking patterns	Prolonged testing	[38,47,89,92]

High variability in sucrose intake versus water intake in sucrose-naïve mice	Neophobia	Habituation to a sucrose solution Sucrose preference as a measure of hedonic sensitivity	[22,38,44,47,85,90-92]

Ceiling values of sucrose preference after massive experience in sucrose ingestion	Sensitization to a sucrose taste	Use of sucrose solution of low concentrations	[24,38,47,87,88,93]

High inter-individual variability in absolute intake of liquids	Inter-individual differences in metabolic needs	Use of a sucrose preference not sucrose intake as a measure of hedonic sensitivity	[25,38,44,47,85,87,92,93]

Studies revealed major sources of confounding factors in the commonly used mouse sucrose preference test. The application of testing conditions which minimize their impact were shown to be benefitial in recently published literature.

Our experiments on mice naive to the taste of sucrose revealed remarkable diversity in animals' reactivity to a sweet taste, which ranged from almost no reaction to excessive sucrose consumption. Repeated exposure to sucrose abolished the first type of behaviour, suggesting that neophobia could underlie this response. A single pre-exposure of mice to concentrated sucrose, a procedure developed in a course of our studies, precluded large variability in their sucrose preference. In different studies, with repeated sessions of the sucrose test, preference and intake of sucrose solutions were found to increase substantially, suggesting that the results of testing in this paradigm depend on the animals' previous experience of sucrose consumption can increase sucrose intake and preference up to ceiling values decreases test's sensitivity [38,47]. This undesirable effect can be counter balanced by application of sucrose solutions of descending concentrations [45].

Our results confirm the findings of others, which demonstrate that in comparison to rats, mice generally demonstrate lower values of sucrose preference and sucrose intake, a pronounced neophobic behaviour during the very first exposure to a sucrose solution, essential inter-individual variability in sucrose preference and, especially, in absolute values of daily liquid intake [11-13,87-93]. Together, the above data show that sucrose preference is a parameter of the sucrose test and is more appropriate than absolute sucrose intake for the analysis of inter-individual differences in hedonic sensitivity in mice. Further, bottle-position preference in mouse drinking behaviour and neophobia together with other factors, may be the cause of essential physiological artifacts in evaluating the sucrose test.

### Correcting for limitations in the sucrose preference test in chronic stress-induced anhedonia

We have undertaken several procedural modifications in order to eliminate the above behavioural artifacts. With our proposed protocol, mice are given a free choice between two bottles for 8-24 hours, one with 1%-sucrose solution and another with tap water; the position of the bottles in the cage is switched halfway through this period. At no point during or prior to this are mice deprived of food or water. To minimize the spillage of liquids during the sucrose test, bottles are filled in advance and kept in an up-side-down position for at least 12 hours prior to testing. In order to balance the air temperature between the room and the drinking bottles, they are kept in the same room where the testing takes place. This prevents liquid leakage resulting from increased air temperature and pressure inside the bottles, when they are filled with liquids which are cooler than the room air.

The consumption of water, sucrose solution and total intake of liquids is estimated simultaneously in the control and experimental groups by weighing the bottles. In order to decrease variability in sucrose consumption during the very first sucrose test (baseline measurement), a day before, animals are allowed to drink a 2.5% sucrose solution in a one-bottle paradigm for 2 hours. The preference for sucrose is calculated as a percentage of the consumed sucrose solution from the total amount of liquid drunk by the formula:

We found that with this method, the error of measurement does not exceed 0.1 ml. This appears to greatly enhance the resolution of the test and more specifically assess hedonic state that takes into account inter-individual variability.

### Submissive traits predict stress-induced anhedonia in C57BL6/N mice

A number of studies have shown that it is possible to predict inter-individual variability, in terms of the stress response, by observing an animal's baseline behaviour. Specifically, animals that show high anxiety [41,60,63], low open field locomotion [30,94], freezing response in averse conditions [42] and decreased exploratory behavior [25,43] will often have a different stress response to their 'normal' littermates. This phenomenon is attracting the attention of researchers and becoming broadly implemented in fundamental and industrial psychopharmacological research. In our experiments, male social behaviour was shown to predict an individual susceptibility to stress-induced anhedonia in mice: individuals with submissive social traits were found to be more vulnerable to the anhedonic state [44-47]. During a 4-week stress induction period, anhedonia was found to occur earlier in all submissive animals. In one study, after only 3.5 weeks, 100% of submissive mice exhibited lowered sucrose preference (< 65%) and matched a given criterion of anhedonia; only 16.6% of aggressive mice that had undergone the same stressors exhibited the criteria for anhedonia at this stage [44]. The ethological analysis of more than thirty parameters of social behavior in a resident-intruder test, performed with our stress model, revealed reduced scores in aggressive and dominance behaviours in mice predisposed to stress-induced anhedonia [95]. A number of studies have shown that animals with subdominant behavioural characteristics will most frequently exhibit low sucrose preference and anhedonia in similar social defeat models [14,21,62,96-99]. The variability in social traits, which is related to the animal's individual ability to cope with environmental stressors, can be considered as a biological factor of species' adaptation and survival. It can be of even higher significance when animals are placed in stressful conditions and, therefore, of evolutionary advantage in general. Recently identified epigenetic molecular mechanisms are suggested to underlie the distinct response of individual animals to environmental challenges [9,94,100].

In our stress paradigm, a resident-intruder test was adapted from the procedure originally proposed by Krsiak and co-authors [101,102]. Initially, the protocol employs a qualitative criterion of differentiation into submissive vs. non-submissive mice, defined as an absence or presence of attacks towards the partner, respectively. In this test, male mice (C57/BL6N - intruders), after being isolated for 3-5 weeks, and when confronted in a neutral cage with another male mouse which had been group-housed (CD1 - residents), will either show aggressive (non-submissive), timid (submissive) or social behaviour. Aggressive activities of both resident and intruder are characterized by attack and aggressive unrest, frequently accompanied by tail rattling. Timid activities consist of alert posture, escape and defence and are never accompanied by aggressive reactions (attacks). Social behaviour includes social sniff and 'climb and follow', this type of social behaviour excludes attacks between the partners; in our study this behaviour was categorized as non-definable with regard to social submissiveness. A manifestation of submissive and aggressive types of social behaviour in C57BL/6N mice was found to appear irrespective of social traits of CD1 counter partners. According to our data, testing procedures did not induce depressive-like behaviours in the forced swim or sucrose preference tests. Group-housing of male C57BL/6N mice is known to result in aggressive behaviour between cage mates that, importantly, enhances a variability in anxiety-related and a number of other behaviours, as well as having a significant impact on the stress response. We therefore chose to test animals from experimental groups, using them as intruders in a resident-intruder test, thus avoiding the undesirable effects of group housing. While it is more usual to analyze the resident animal in this paradigm, we believe that our protocol of testing social behaviour allowed us to preclude major artifacts in comparing animals from anhedonic and non-anhedonic groups.

**Figure 2:**
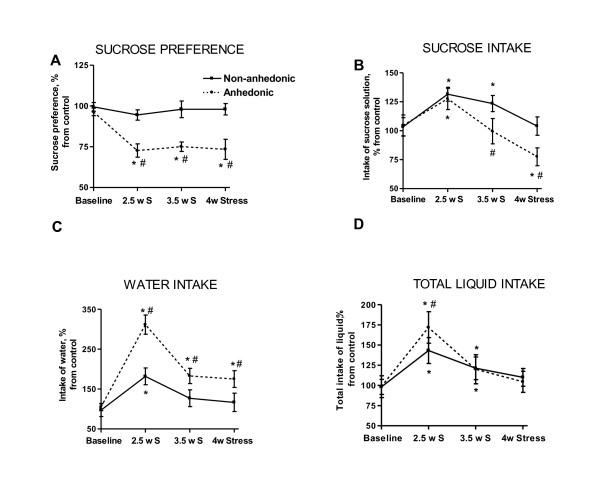
**Differential stress-induced changes in the sucrose test parameters in anhedonic and non-anhedonic mice**. **(A) **Sucrose preference in the anhedonic group is significantly lower than in non-anhedonic and control mice after 2.5, 3.5 and 4 weeks of stress. **(B) **Sucrose intake in the anhedonic group is significantly increased after 2.5 weeks of stress and significantly decreased after 3.5 weeks of stress (vs. non-anhedonic group) and after 4 weeks of stress (vs. control and non-anhedonic group). Non-anhedonic mice show elevated sucrose intake after 2.5 and 3.5 weeks of stress. **(C) **Water intake is elevated in the anhedonic animals after 2.5 - 4 weeks of stress (vs. control and non-anhedonic group). In the non-anhedonic group; water intake is increased after 2.5 weeks of stress as compared to control. **(D) **Total liquid intake is elevated both in the anhedonic and in non-anhedonic animals after 2.5 and 3.5 weeks of stress (vs. control group). After 2.5 weeks, anhedonic mice show significantly higher total liquid intake than non-anhedonic mice. Parameters of the sucrose test are expressed as a percentage of the mean values of the control group, and compared between anhedonic (dashed line) and non-anhedonic (plain line) groups during a 4-week stress procedure as mean ± (SEM) (*p < 0.05 vs. control group; #p < 0.05 vs. non-anhedonic group; Mann-Whitney).

Interestingly, in populations with an initially low percentage of submissive animals (15-20%), all submissive mice develop anhedonia. In populations with a high percentage of submissive individuals (>50%), the percentage of non-submissive (dominant or aggressive) animals susceptible to anhedonia is significantly lower in comparison to a submissive cohort of mice. Remarkably, social characteristics of animal batches, such as a percentage of aggressive mice, were found to be related to behavioural patterns during stress. For example, changes in parameters such as sucrose intake and preference, and total liquid intake during different phases of the course of stress exposure were observed over a several years in our lab, under identical experimental conditions [39,47]. For instance, in populations with an initially low percentage of submissive animals, statistically significant decreases in sucrose preference in the stressed group occur at 3.5 weeks after introduction of the stressor. Interestingly, in these animals, stress exposure causes an increase in sucrose intake and preference at the beginning of the stress procedure, and leads to a slight elevation of water intake. In contrast, in experimental groups containing high percentage of submissive individuals (>50%) the drop in sucrose preference is detected much earlier, after 2.5 weeks of stress; these animals demonstrate an increase in sucrose intake and sharp elevation of water consumption [Figures 1 and 2B; [44,48]]. Over our 6 years of studying them, the C57BL/6N strain of mice have shown a remarkable range, from 15-85%, in the proportion of individuals with submissive social characteristics.

Taken together, these data suggest that initial populations with unbalanced social traits could lead to different susceptibilities in terms of the chronic stress model, and may be one of the sources of unstable reproducibility. Our studies have shown that, in terms of development of anhedonia, no differences in initial open field, exploratory rearing, step-down avoidance, novel object or anxiety-like behaviours were exhibited between mice from anhedonic and non-anhedonic groups [38,39,44-49]. These data are not always consistent with results published by other groups, which, for example, found a correlation between initial elevated anxiety-like behaviour and enhanced stress-response [41,63]. The discrepancy between this and our data could be explicated by differences in substrains used and protocols of testing anxiety-like behaviours. Others, however, have largely supported our findings [21] by showing a lack of differences in various behavioural parameters and baseline sucrose intake and preference between animals from anhedonic and non-anhedonic groups. We believe that in general, balancing of percentage of animals upon a parameter, which is predictive for individual susceptibility to a depressive-like state in any particular chronic stress depression model, can greatly help increase its reliability, especially when the testing of potential anti-depressant treatment is involved.

### Identifying and compensating for behavioural artifacts caused by stress-induced hyperlocomotion

Hyperlocomotion as an increase of a distance traveled by an animal over a time span is not uncommonly seen in chronic stress experiments. On another hand, many studies using chronic stress models of depression showed abnormal changes in rodents' behavior, which contradict well documented profiles of states of stress and depression in animals [13,14]. These changes are often called "paradoxical" or "anomalous" since they are opposite to the general known patterns of behavioural inhibition, impaired coping and increased scores of anxiety observed in patients with stress-related disorders and respective pre-clinical paradigms. For example, the chronic mild stress procedure was shown to not affect or to increase anxiety-like behaviours, i.e. time spent in anxiety-related areas of the elevated plus maze and dark/light box and to reduce immobility time in forced swimming tests in mice [103-107]. Exposure to a chronic stress was shown to lead to increased ambulation in the open-field test in mice [28]. Taking into account the aforementioned data from the literature, as well as our own results, which demonstrated drastic increases in speed and total path in the open-field, anxiolytic-like changes in the dark/light and elevated O-maze paradigms alone with reduced duration of floating in the forced swim test in chronically stressed mice (Table 7), we suggest this hyperlocomotion is an non-specific consequence of chronic stress and is triggered by the stressful procedure of testing. Thus, reduction of the stress impact of testing conditions, e.g., diminishing light intensity should be considered as an important pre-requisite of behavioral analysis in the chronic stress models. These findings were implemented in our behavioural protocols in which we were using "mild" conditions of testing with low stress impact [108]. We believe this was a key factor in enabling us to identify consistent behavioural correlates of anhedonia in our model.

**Table 7:** Identifying and compensating for behavioural artifacts caused by stress-induced hyperlocomotion

	Anxiety-like behavior		Floating in FST		Open field locomotion	
	
	Standard protocol	Mild protocol	Standard protocol	Mild protocol	Bright/modest lighting	Weak lighting
**Short stress**	↑	↑	↔	↔	↔	↔

**Chronic stress**	↓	↑	↓	↑	↑	↔

**Chronic stress + diazepam**	↑		↑		↔	

Reduction in illumination, application of "mild" testing conditions or bolus diazepam injection preclude hyperlocomotion and "anomalous" behaviour in anxiety and forced swim tests in chronically stressed mice. Mice subjected to short stress (1 week duration) do not exhibit "anomalous" behaviour. Arrows indicate an increase in the parameter (↑), a decrease (↓), or lack of significant changes in comparison to non-stressed control mice (↔).

Recent studies described similar phenomenon in several laboratory mouse strains [109-111]. While various effects of chronic stress on general locomotion in rodents were described [34,96,112-1115], lighting conditions employed during testing were reported to be a significant factor of general activity in the stressed animals [116-118]. We believe that stress-induced hyperactivity is a typical phenomenon in chronically stressed C57BL/6N mice and is potentially a major source of artifacts in behavioural analysis of chronic stress data and that this can explain previously reported contradictions resulting from similar paradigms.

### Increased liquid intake and home-cage locomotion in anhedonic versus non-anhedonic mice: indication of an elevated stress-response?

Stress-induced increases in sucrose and water intake in mice and rats exposed to a prolonged stress have been documented elsewhere [21,34,119,120]. Parallels have been made in the literature between these signs of elevated consummatory behaviour in stressed animals and other indicators of behavioural invigoration, e.g. enhanced swim scores, excessive grooming, increased activity in anxiety paradigms and in other tests [121-123]. Apart from general behavioural invigoration and an increase in consumption scores, the augmentation of general liquid intake observed in chronic stress paradigms is believed to result from a stress-induced polydipsia, increased metabolic needs, diabetes mellitus and altered hormone secretion from both the hypothalamus and hypophysis [21,124,125]. Our studies revealed drastic changes in water and sucrose solution consumption as well as home cage locomotion during the course of the chronic stress procedure in C57BL/6N mice. Analysis of animals from the anhedonic and non-anhedonic groups at different phases of stress, and after its termination revealed distinct patterns of dynamics within these parameters [39,44,45,47].

**Figure 3:**
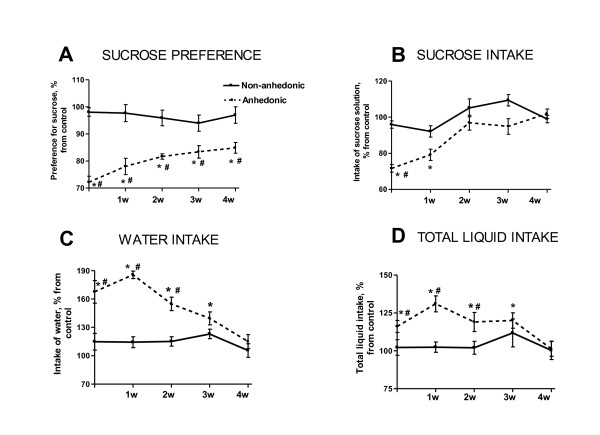
**Dynamics of the sucrose test parameters recovery in anhedonic and non-anhedonic mice**. Parameters of the sucrose test were expressed as a percentage of mean values of the control group, and compared between anhedonic (dashed line) and non-anhedonic (plain line) groups during a 4-week stress procedure (*p < 0.05 vs. control group; # p < 0.05 vs. non-anhedonic group; Mann-Whitney). **(A) **Sucrose preference in the anhedonic group is significantly lower than in the non-anhedonic and control mice throughout the entire experiment. **(B) **Sucrose intake in the anhedonic group is significantly decreased after the termination of stress (vs. control and non-anhedonic group) and during week 1 of the stress-free period (vs. control group). **(C, D) **Water consumption and total liquid intake are elevated in the anhedonic animals up to 3 weeks after termination of stress as compared to control, and up to 2 weeks when compared to non-anhedonic mice. Data are expressed as mean ± (SEM).

Our extensive experience with the chronic stress model of depression has repeatedly shown that anhedonic mice develop increased sucrose preference and intake between days 14-21 of a 28 day paradigm. This effect is pronounced and of significant duration in anhedonic animals but is transient in non-anhedonic groups (Figure 2, adapted from [45]). Similarly, total liquid intake is also increased in both groups but anhedonic animals maintain this change over a longer period than their non-anhedonic littermates. The increased water intake in anhedonic animals lasts for several weeks after the termination of chronic stress; non-anhedonic animals, however, exhibited normal liquid consumption during the after-stress period (Figure 3; adapted from [45]). Of note, chronic administration of antidepressants citalopram and imipramine, applied during and after chronic stress, reduced stress-induced increase in water consumption in our model [45].

Increased intake of liquids in mice from the anhedonic group was paralleled in our paradigm by sharp increases in home-cage horizontal activity, which was not observed the non-anhedonic group [47]. The latter behaviour was studied using the System and software for Automatic Measurement of Animal Behaviour (SAMAB), where mice were housed individually in specialized cages with infrared detection of horizontal movement.

**Figure 4:**
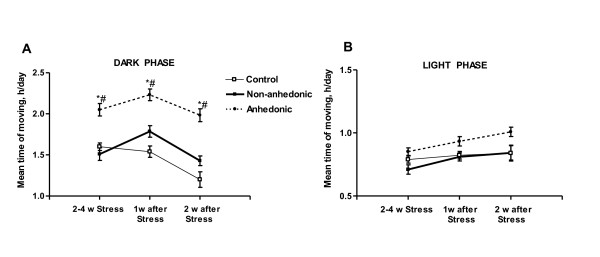
**Anhedonic mice display lasting increases in home cage activity during the dark phase of the day**. **(A) **During the dark phase of the diurnal cycle, mean time of horizontal movement of the anhedonic group (dashed line) is significantly elevated during 2nd-4th weeks of stress, and during weeks 1 and 2 after the termination of the stress procedure, as compared to the non-anhedonic (plain thick line) and control (plain thin line) groups (*p < 0.05 vs. control group and # p < 0.05 vs. non-anhedonic group; Mann-Whitney). This parameter does not change in the non-anhedonic group throughout the entire experiment (p > 0.05 vs. control group). **(B) **During the light phase of the day, no difference between the groups in home cage activity is observed (p > 0.05). Data are expressed as mean ± (SEM).

Mean duration of movement was significantly elevated, in anhedonic animals only, during the dark phase of the day/night cycle between days 14 and 21 during the stress procedure and for at least 14 days after termination of the procedure (Figure 4A); no differences between the groups are observed during the light phase (Figure 4B).

While the nature of the elevated intake of palatable solutions during chronic stress is unclear, several reports suggest that consumption of sweetened solutions can evoke an antidepressant-like effect in rodents [13]. Interestingly, mice from a group susceptible to stress-induced social avoidance/anhedonia were shown to have higher scores of conditioned place conditioning in comparison to resilient mice [21]. This led to speculation that enhanced sucrose intake and preference in chronically stressed animals might be "adaptation" to stress at its early stages. As such, their increase in mice from an anhedonic group might reflect a "hyperadaptation" to stress in this population, and that the development of anhedonia at the late stage of stress can manifest itself as a state of a "distress" in this subgroup.

Both pronounced elevation of water intake and enhanced home cage locomotion in anhedonic animals may result from general sympathetic activation, induced by chronic stress, and thus, reflect a pronounced response to stress in these animals [21,126]. Our results also suggest that stress-induced anhedonia in the current mouse model is accompanied by an altered pattern of the day/night activity, which correlates with compromised sleep-wake patterns in depressed patients [127], and which is not seen in stressed animals without hedonic deficit. These data are in line with other studies which found stress-induced hypertherimia and a decrease in circadian amplitude in a subgroup of mice with a depressive-like state in a social defeat model [21]. Together, differential patterns of liquids intake and home-cage locomotion in anhedonic versus non-anhedonic mice may be reflective of a higher stress susceptibility in the first cohort of animals.

### The effects of citalopram in anhedonic and non-anhedonic mice

**Figure 5:**
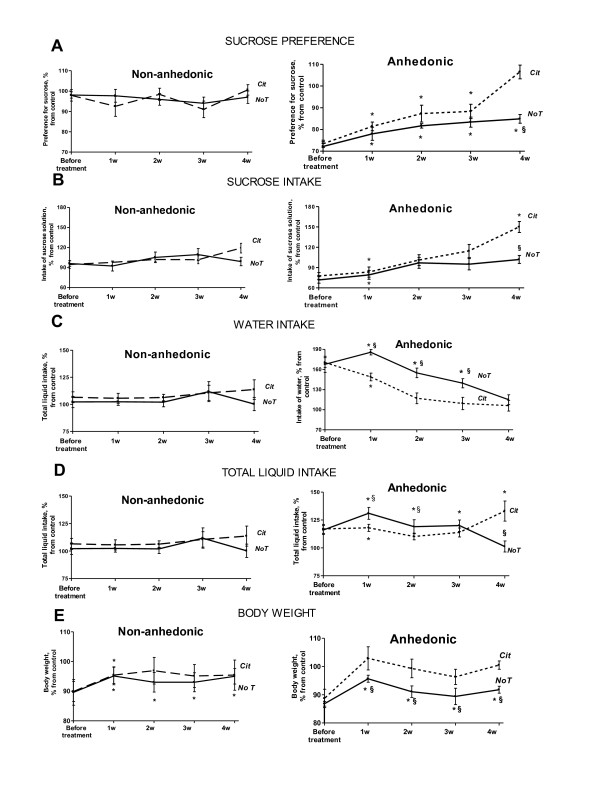
**Effects of citalopram on sucrose test parameters and body weight in anhedonic and non-anhedonic mice**. **(A-E) **Data are expressed as a percentage of the control and presented as medians ± interquartile intervals. Plain lines indicate non-treated groups, dashed lines indicate citalopram-treated groups. **NoT**: non-treated group; **Cit**: citalopram-treated group (*p < 0.05 vs. control group; § p < 0.05 vs. citalopram-treated anhedonic mice).

Recent studies suggest that chronic SSRI treatment in mice resilient and susceptible to social defeat stress exerts distinct physiological and molecular effects on the ventral tegmental area and the medial prefrontal cortex [23,53,54,128,129]. In line with these findings, pharmacological validation of a proposed model of stress-induced anhedonia with a "post-stress" administration of citalopram demonstrated its differential effects in anhedonic and non-anhedonic groups. In one of the studies, exposure of animals to chronic stress was followed by citalopram delivery via the drinking solution (15 mg/kg/day) for four weeks. Sucrose preference and body weight were monitored weekly in control, anhedonic and non-anhedonic mice [45]. Sucrose intake and preference increased in citalopram-treated, but not non-treated anhedonic mice on the 4th week of the dosing schedule; non-anhedonic and control mice showed no changes in sucrose preference at any time point (Figure 5A, adapted from [45]). Multiple regression analysis showed that restoration of a preference for sucrose in the anhedonic group was mostly due to a several fold elevation of sucrose ingestion, a phenomenon typical for prolonged treatment with antidepressants of various classes [11-14]. Interestingly, while at this time point of the experiment, mean sucrose consumption in the non-treated anhedonic group was not different from the values of non-treated control and non-anhedonic groups, citalopram essentially affected sucrose intake exclusively in anhedonic animals (Figure 5B). In the forced swim test, anhedonic mice treated with an antidepressant, demonstrated a decrease in duration of floating behaviour, which was elevated in the non-treated anhedonic group. Of note, chronic administration of citalopram did not affect floating of the non-anhedonic animals and non-significantly elevated this parameter in non-stressed control mice. Together, these data suggest that the occurrence of a depressive-like state is a pre-requsite of animals' sensitivity to the action of antidepressants in a chronic stress paradigm. More studies are required to elucidate the physiological basis of selective effects of citalopram on depressive-like features in stressed anhedonic mice.

Experiments with various methods of citalopram administration indicated that in anhedonic animals, chronic administration of drug reduced water intake, which was enhanced in this group of mice, and did not affect this parameter in the non-anhedonic animals (Figure 5C). Interestingly, anhedonic animals from the citalopram-treated stressed group had a higher average body weight than non-anhedonic animals from the same group (Figure 5E). Citalopram restored the body weight of anhedonic mice after the 1st week of post-stress treatment but did not have such an effect in non-anhedonic animals. During weeks 2-4, body mass of the latter group was lower, although not significantly, than in control and citalopram-treated anhedonic aniamls. Prior to citalopram treatment, both anhedonic and non-anhedonic animals exhibited similar patterns of weight loss compared to controls. Restoration of body weight by antidepressant treatment, particularly citalopram, in animal models of depression has been shown to to accompany recovery from a depressive-like state [130]. The distinct effects of citalopram on body weight in anhedonic and non-anhedonic mice may be related to the metabolic differences between these animals: repeated experiments with our model have revealed a general higher baseline body mass in anhedonic than in non-anhedonic animals (Strekalova, *unpublished resu*lts). These results suggest that citalopram is capable of altering a number of variables in anhedonic animals. This provides more refined analysis of the effects of antidepressant treatment with respect to the states of stress and anhedonia.

## Conclusions

In line with available literature, our studies identified distinct physiological and molecular profiles of anhedonic and non-anhedonic groups of mice subjected to stress. As such, the proposed mouse paradigm and other models which enable the segregation of subpopulation of animals with and without a depressive-like pattern can be a tool for addressing the biology of individual susceptibility and resilience to depression. Besides that, this approach provides other valuable advantages in modelling of depressive disorder in animals, as for instance, it allows the differentiation between core and subsidiary depressive features and let to simulate the co-morbidity of depression and other stress-related disorders, e.g., anxiety diseases [78,79,131,132]. This opens new possibilities in pre-clinical studies aimed at the differentiation between therapeutic effects of antidepressants to depression symptoms and other concominant pathological changes. We believe that data on distinct sensitivity to an antidepressant treatment of stressed mice with and without depressive-like syndrome argue for the validity of our paradigm in mimicking such clinical aspects of the disorder.

We feel the importance of emphasizing the fact that the use of internal control in a chronic stress depression paradigm and behavioural assessment of the validity of its definition became possible only when the methodology of chronic stress model in tested strain of mice was essentially modified. These modifications mostly concern (1) the sucrose test protocol in mice, the accuracy of which could be sharply increased to allow distinguish inter-individual differences in the occurrence of signs of anhedonia, and (2) the identification and overcoming of confounds in behavioral testing related to stress-induced hyperlocomotion. Another feature which appeared to be important for more accurate group comparison with the chronic stress model is their balancing upon (3) a percentage of individuals with submissive and dominant social traits, which predicted a susceptibility to a stressed-induced anhedonia in employed strain.

Together, results discussed here argue for the use of a subgroup of individuals who are negative for the induction of a depressive phenotype with experimental paradigms of depression as an internal control, for more advanced modeling of this disorder in animals.

## Competing interests

The authors declare that they have no competing interests.

## Authors' contributions

TS carried out behavioral studies, drafted the manuscript and coordinated manuscript preparation. YC helped with the molecular genetic studies and drafted the manuscript. NK participated in the analysis of gene expression studies and helped to draft the manuscript. MB took part in the gene expression data analysis. DM performed the treatment of raw gene expression data and statistical analysis. PL carried out IPA analysis of gene expression data and drafted the manuscript. HS participated in the design of the studies and its coordination and helped to draft the manuscript. All authors read and approved the final manuscript.
